# Brexit and What It Means for Global Health

**DOI:** 10.4269/ajtmh.17-1000

**Published:** 2018-02-05

**Authors:** Brian Greenwood

**Affiliations:** Faculty of Infectious and Tropical Diseases, London School of Hygiene & Tropical Medicine, London, United Kingdom

The results of a referendum held in the United Kingdom (UK) on June 23, 2016 in which 33 million voters decided by a margin of 51.9–48.1% to leave the European Union (EU) came as a shock to many in the UK who had been confident that this would not be the case, and was accompanied by a sense of disbelief among many outside the UK. Subsequent analysis has shown that the “leave” vote was highest in the oldest age groups, those with less formal education, and in areas outside the main metropolitan centers; young voters and residents of cities with a strong academic center were strongly in favor of “remain.” The vote provided a binary choice of leave or not, with no opportunity to consider the kind of arrangements that would follow a decision to leave. In the 18 months after the referendum, protracted discussions have taken place between the UK government and the European Commission on the nature of Brexit. Some progress has been made in these discussions on the “divorce bill,” the status of European citizens resident in the UK, and vice-versa, and on how the border between Northern Ireland and the Republic of Ireland, which will remain in the EU, will be managed. Negotiations will now move onto broader issues such as trade, and these negotiations will include discussions on the future relationship between the UK and the EU on science and innovation. However, it is still not clear whether a final agreement will be reached before the UK leaves the EU on March 29, 2019 and, if an agreement is reached, what form this might take. If an agreement is reached, it is likely that some kind of transition period will be needed to allow the new arrangements to be implemented.

The outcome of the Brexit referendum will have major consequences for the UK in many areas, including health and science. The UK National Health Service is very dependent on contributions of doctors and nurses from EU countries, and recruitment of staff from the EU has already fallen substantially. The decision to move the European Medicines Agency, Europe’s regulatory agency, from London to Amsterdam could influence major pharmaceutical agencies regarding investment in the UK, including agencies involved in developing medicines for the developing world. The decision to leave is a major concern for UK scientists because it is now unclear whether they will in future be able to access research funds provided by the EU, a major source of financial support for many UK universities and research institutions, and whether they will be able to continue to recruit talented European researchers to their research groups.

Should the Brexit decision be of any interest or concern to readers of the *American Journal of Tropical Medicine and Hygiene* who live outside the UK? I believe that it should for several reasons. First, the decision to leave the EU may impair the future ability of UK scientists to work effectively in partnership with colleagues in the EU and elsewhere in many areas of science, including global health, a field in which UK scientists currently play an important role. The UK government has promised to cover the costs of existing grants provided by EU institutions after departure from the EU, but how UK scientists will be able to participate in EU supported research projects after that date remains uncertain. For example, it is uncertain whether UK scientists will be eligible to apply for funding from the European Developing Countries Clinical Trials Partnership, an important source of support for collaborative research and research capacity development projects linking European scientists and those from developing countries. Second, the Brexit decision could have a direct impact on funding for global health. Currently, the UK is one of the most generous donors to international organisations concerned with global health such as the Global Fund and the Global Alliance for Vaccination and Immunization, and a strong supporter of public–private partnerships involved in the development of products for the developing world such as the Medicines for Malaria Venture. Although there is no certainty over the financial impact of Brexit on the UK economy, and this will depend to some extent on the nature of the final deal, most of the financial experts reckon that there will be a downturn in the UK economy after Brexit, at least in the short term. Currently the UK government is providing substantial financial support to national research on global health issues through a Global Challenges Research Fund, but should Brexit lead to a downturn in the overall economy, the government will have less money overall to spend and may change its priorities to issues of more local concern. Finally, the leave vote suggests that there has been a change in attitude among a significant proportion of the population of the UK toward a more parochial view of the place of the UK in the world and away from the idea that a wealthy country such as the UK should be making a major contribution to meeting the challenges that threaten the world as a whole such as global warming, food security, migration, and tropical infectious disease, a change in attitude also apparent in some other western democracies including the USA. The success of the Brexit “leavers” could encourage those with similar views in other countries within the EU and elsewhere to press for a similar change in direction.

A high proportion of UK scientists were against leaving the EU, especially those involved in global health, but after a period of initial shock and a lot of whinging, there has now been recognition that unless there is some completely unexpected turn of events, the UK is on course to leave the EU on March 29, 2019. Consequently, efforts are now being made by the UK’s scientific community to try to mitigate the potentially damaging impact of Brexit on UK science and to maintain the ability of UK scientists to contribute to international challenges. Led by institutions such as the Royal Society, the Wellcome Trust, and major academic institutions, including my own, the government has been intensively lobbied to ensure that arrangements for easy movement of scientists between the UK and the EU is given prominence in the ongoing negotiations. Another option that is being explored by UK universities and research institutions is establishment of partnerships with EU institutions that will allow UK institutions to access EU research funds, but it is unlikely that this will be acceptable to the EU unless these are true partnerships and not just paper exercises. Ensuring that global health is not neglected should Brexit lead to a significant financial downturn in the UK will be a major challenge, especially considering an increasing skepticism concerning the value of overseas aid. Ensuring continuing UK government support for national global health activities and for major international organisations for which the UK is a strong financial supporter will require a committed effort from UK scientists, a task that could be helped by support from academic institutions in the EU and more widely.

The decision of the UK to leave the EU has been a bad one for UK science overall, potentially reducing its ability to contribute to research on issues related to global health and also to sustain its major development programs. However, imaginative ways are being explored to meet these challenges and to ensure that the UK can continue to collaborate with its partners in the EU and elsewhere to continue to make a major contribution to global health.

